# Assessing the impacts of climate change on the potential distribution of the medicinal plant *Bidens biternata* in China using Biomod2

**DOI:** 10.3389/fpls.2026.1798675

**Published:** 2026-05-13

**Authors:** Yi Liu, Pengfei Zhang, Hong Zhang, Wei Gao, Han Luo, Shoujin Liu, Jiangmiao Hu

**Affiliations:** 1 College of Pharmacy, Anhui University of Chinese Medicine, Hefei, China; 2 Anhui Provincial Testing Institute for Food and Drug Control, Hefei, China; 3 Dexing Research and Training Center of Chinese Medical Sciences, Dexing, China; 4 Jiangxi Province Key Laboratory of Sustainable Utilization of Traditional Chinese Medicine Resources, Institute of Traditional Chinese Medicine Health Industry, China Academy of Chinese Medical Sciences, Nanchang, China; 5 Institute of Traditional Chinese Medicine Resources Protection and Development, Anhui Academy of Chinese Medicine, Hefei, China; 6 China Resources Sanjiu (Lu’an) Traditional Chinese Medicine Industry Development Co., Ltd., Lu’an, China; 7 Kunming Institute of Botany, Chinese Academy of Sciences, Kunming, China

**Keywords:** *Bidens biternata*, Biomod2, climate change, habitat suitability, species distribution modeling

## Abstract

**Introduction:**

Climate change is expected to reshape the potential distribution of medicinal plants, with direct implications for conservation, cultivation zoning, and sustainable utilization. This study aimed to assess the current and future habitat suitability of *Bidens biternata* in China under climate change.

**Methods:**

Using the biomod2 R package (v4.2-6), we developed an ensemble species distribution model based on 493 raw occurrence records compiled from field surveys, GBIF, and CVH, which were spatially thinned to 197 independent records at a 5-km grid. After collinearity screening, 15 environmental predictors were retained. Twelve algorithms were calibrated using 1,000 pseudo-absence points, and future suitability was projected under SSP126, SSP245, SSP370, and SSP585.

**Results:**

The committee-averaging ensemble model showed high predictive performance, with TSS = 0.842 and AUC = 0.96. Temperature-related predictors, especially bio2, bio3, and bio8, dominated model performance. Under the current climate baseline, highly suitable habitat was mainly concentrated in monsoonal eastern and southern China and parts of Southwest China, covering 2.68 × 10^5^ km^2^ (17.20%). Future projections indicated an expansion of highly suitable habitat during 2021–2080, followed by late-century divergence under higher-forcing pathways, with broader marginally suitable belts and reduced highly suitable fractions. Centroid trajectories indicated an overall northward to northwestward shift, whereas MESS/MoD diagnostics distinguished persistent suitability cores from climatically novel expansion fronts.

**Discussion:**

These findings identify more reliable conservation and cultivation priority areas in the middle-lower Yangtze region, South China, the Sichuan Basin, and parts of Southwest China. Northern frontier zones should be treated as validation and trial-introduction areas rather than immediate large-scale production bases.

## 1 Introduction

Climate change is widely recognized as a major driver of species-range dynamics because it alters temperature and precipitation regimes, increases the frequency and intensity of extreme events, and reshapes the ecological processes that determine habitat suitability (Chen et al., 2011; Dieleman et al., 2015). For plants, especially taxa with relatively narrow climatic tolerances or limited dispersal capacity, ongoing warming and shifts in hydrothermal conditions can lead to range contraction, expansion into newly suitable areas, and redistribution toward higher latitudes or elevations (Wang et al., 2021; Guan et al., 2024). These responses have been documented across biomes and are expected to intensify throughout the twenty-first century (Sun et al., 2026). In China, steep transitions from tropical to temperate climates and from humid monsoonal regions to arid inland areas create pronounced hydrothermal gradients. Because many plant distributions are organized close to these environmental limits, even modest climatic shifts can alter the boundaries of suitable habitat, especially at range margins (Zhang et al., 2014; Feng et al., 2023).

Medicinal plants represent a distinctive component of biodiversity because their value is jointly shaped by ecological suitability and human-use systems (Yue et al., 2022; Chen et al., 2026a). Climate-driven range shifts may alter the availability and conservation status of wild resources (Dieleman et al., 2015), disrupt cultivation and supply chains, and reconfigure suitable cultivation zones (Liu et al., 2025). In addition, climatic conditions such as temperature, precipitation, solar radiation, and seasonality can influence the biosynthesis and accumulation of bioactive metabolites (Farsi et al., 2023), potentially affecting the quality and efficacy of herbal materials (Manukyan, 2019). Therefore, quantifying climate-induced changes in potential distributions is critical for anticipatory conservation (*in situ* and *ex situ*) and for adaptive planning of medicinal-plant cultivation and regional industry development (Shan et al., 2023; Zaman et al., 2025).

Species distribution models (SDMs), also termed ecological niche models, relate occurrence records to environmental predictors to estimate habitat suitability and potential geographic distributions (Feng et al., 2024; Oeser et al., 2024; Serafini et al., 2025). Because they provide a quantitative basis for identifying current suitable habitat, potential refugia, and climate-driven redistribution, SDMs are now widely used in biodiversity conservation, invasive-species risk assessment, and medicinal-plant planning under climate change (Naughtin et al., 2025). However, projections from a single algorithm can be sensitive to assumptions about response shapes, predictor selection, prevalence, and sampling bias (Safaei et al., 2021). Ensemble forecasting approaches that integrate multiple algorithms can reduce algorithm-specific uncertainty and are increasingly recommended (Hao et al., 2020). The biomod2 framework provides an integrated R workflow for multi-algorithm SDM development and ensemble prediction (Gu et al., 2024), including repeated cross-validation, model evaluation (e.g., AUC and TSS), and weighted or committee-based aggregation of model outputs (Di Cola et al., 2017; Mu et al., 2025).

**Figure 1:**
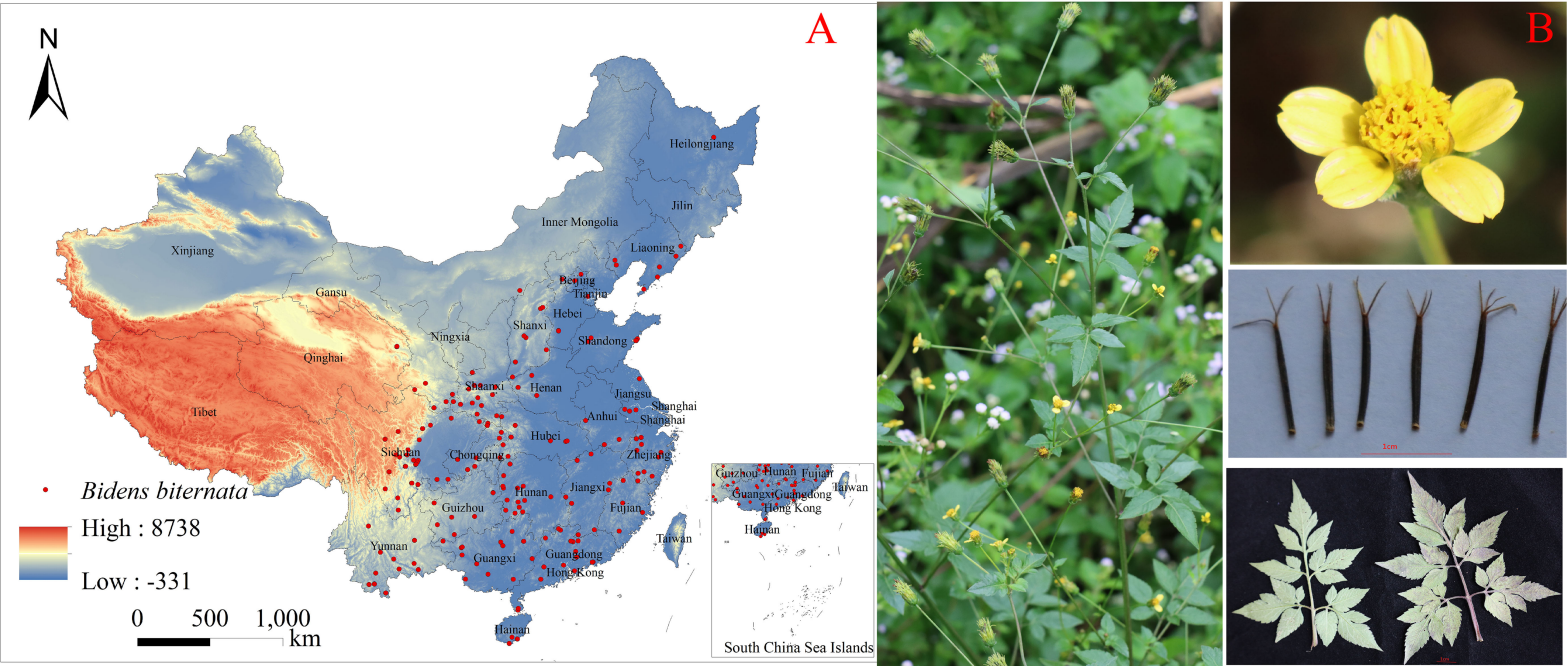
**(A)** Distribution sampling site of *B.biternata* in China. **(B)** Representative image of *B.biternata.*.

*Bidens biternata* (Lour.) Merr. et Sherff. (Asteraceae), commonly known as Jinzhanyinpian, is an annual herb used in traditional medicine in China (Zahara et al., 2019). It is characterized by erect stems 30–150 cm tall, bipinnately divided leaves, capitula 7–10 mm in diameter with or without yellow ray florets, and linear four-angled achenes; flowering usually occurs from September to November (**Figure 1**). The species is native to China and is distributed widely in South, East, Central, and Southwest China, with additional records in northern provinces such as Hebei, Shanxi, and Liaoning (China, 2007). The whole plant is traditionally used for heat-clearing and detoxifying and has been used clinically for conditions including upper respiratory tract infections, sore throat with swelling and pain, acute appendicitis, and acute icteric hepatitis (Kinuthia et al., 2016). *B.biternata* is also an ingredient in multiple Chinese patent medicines (e.g., Ganmaoling and Sanjin Ganmao Pian). Although the species is widely distributed in China, national-scale, multi-scenario assessments of its potential distribution under CMIP6 climate pathways remain limited (Wani et al., 2025).

Here, we address three questions: (1) Which environmental variables primarily shape the current distribution of *B. biternata* in China? (2) How will the area and spatial configuration of suitable habitat change across future scenarios and time horizons? and (3) Where are potential refugia and emerging expansion zones, and how consistent are projected shifts in direction and magnitude? We compiled occurrence records from field surveys and databases, reduced sampling bias via spatial filtering, selected a low-collinearity predictor set, and developed an ensemble SDM using Biomod2 (Gan et al., 2025). We then projected suitability under multiple SSPs and periods and quantified changes in suitable area (Guo et al., 2025), centroid displacement, and climatic novelty using MESS/MoD diagnostics to support conservation and cultivation planning.

This study contributes beyond routine distribution mapping in three ways. First, it uses a multi-algorithm Biomod2 ensemble rather than a single model, thereby reducing algorithm-specific uncertainty. Second, it evaluates multiple CMIP6 SSP pathways and time horizons to quantify not only area change but also spatial redistribution of habitat suitability. Third, by combining centroid tracking with MESS/MoD diagnostics, it identifies both potential climatic refugia and climatically novel expansion fronts, providing a more decision-oriented basis for conservation and cultivation planning for medicinal plants.

## 2 Materials and methods

### 2.1 Species occurrence data

We compiled occurrence records of *B. biternata* from field surveys across Jiangsu, Anhui, Jiangxi, Henan, Hubei, Sichuan, Guizhou, Yunnan, and Shaanxi Provinces, supplemented with georeferenced records from the Global Biodiversity Information Facility (GBIF; https://www.gbif.org/) and the Chinese Virtual Herbarium (CVH; http://www.cvh.ac.cn/). In total, 493 raw occurrence records were assembled, including 46 from field surveys, 382 from GBIF, and 65 from CVH; duplicate, incomplete, and spatially inconsistent records were removed before modeling. To mitigate spatial sampling bias and reduce spatial autocorrelation, we thinned occurrences to the resolution of the environmental predictors (5 km × 5 km), retaining at most one record per grid cell. After thinning, 197 spatially independent records were retained for modeling (**Figure 1**).

### 2.2 Environmental variables

**Figure 2:**
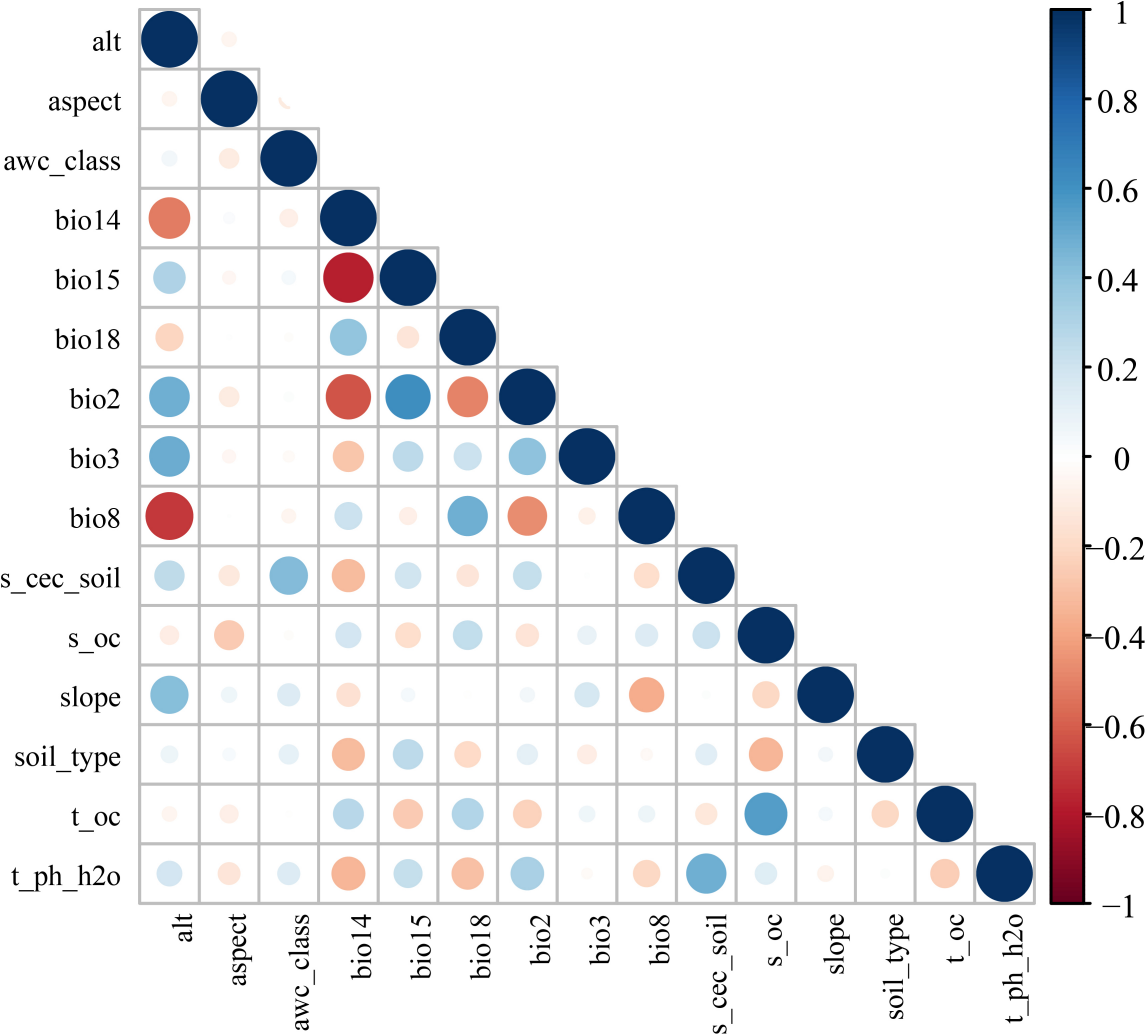
Pearson correlation matrix of candidate environmental predictors used for collinearity screening before model calibration.

We assembled 19 historical bioclimatic variables (1970–2000; 2.5 arc-min resolution) from WorldClim (Fick and Hijmans, 2017). Topographic variables (elevation, slope, and aspect) were derived from a digital elevation model (DEM; 500 m resolution) obtained from the Geospatial Data Cloud (Pizarro et al., 2025). Soil variables (e.g., soil organic carbon, soil pH, cation exchange capacity, soil type, and available water capacity) were obtained from the Harmonized World Soil Database (Gray et al., 2011). For future projections, we used CMIP6 climate outputs from the BCC-CSM2-MR general circulation model under four Shared Socioeconomic Pathways: SSP126 (low forcing/sustainability pathway), SSP245 (intermediate stabilization pathway), SSP370 (medium-high forcing pathway), and SSP585 (high forcing/fossil-fueled development pathway) (Yazdandoost et al., 2021; Liu et al., 2022). BCC-CSM2-MR was selected because it has shown reasonable skill in reproducing temperature and precipitation patterns over China and has been widely used in regional climate assessments for China (Liu et al., 2022).All raster layers were resampled, aligned, and clipped to a common grid consistent with the occurrence-thinning resolution (Das et al., 2020). To reduce linear multicollinearity among predictors, we calculated pairwise Pearson correlation coefficients and removed one variable from each highly correlated pair (|r| ≥ 0.8; **Figure 2**). Pearson’s r was used here to screen linear redundancy among gridded predictors rather than to perform inferential statistical testing; therefore, a formal normality test was not required. Although Spearman’s rank correlation can also be used in SDM studies, Pearson screening is widely applied for continuous environmental rasters when the goal is to remove collinear predictors before model calibration. The final set of 15 predictors was then used for all replicated runs and ensemble projections (**Table 1**).

**Table 1:** Selected environmental predictors retained for modeling the distribution of *B. biternata*.

Code	Environment variables	Unit
alt	Altitude	m
aspect	Aspect	–
awc_class	Available water capacity	%
bio14	Precipitation of the driest month	mm
bio15	Precipitation seasonality	mm
bio18	Precipitation of the warmest quarter	mm
bio2	Mean diurnal temperature range	°C
bio3	Ratio of diurnal temperature range to annual temperature range	–
bio8	Mean temperature of the wettest quarter	°C
s_cec_soil	Subsoil cation exchange capacity	cmol/kg
s_oc	Subsoil organic carbon content	%weight
slope	Slope	°
soil_type	Soil type	–
t_oc	Topsoil organic carbon content	%weight
t_ph_h20	Topsoil pH	-log(H+)

### 2.3 Model calibration

Species distribution modeling was performed in Biomod2 R package (v4.2-6), an ensemble platform for species distribution modeling (Ma et al., 2025), using 12 algorithms: artificial neural networks (ANN) (Kaviani and Sohn, 2021), classification tree analysis (CTA) (Giraldo and Kucuker, 2025), flexible discriminant analysis (FDA) (Hallgren et al., 2019), generalized additive models (GAM) (Uusitalo et al., 2019), generalized boosted models (GBM) (Soilhi et al., 2024), generalized linear models (GLM) (Babu et al., 2023), multivariate adaptive regression splines (MARS) (Giraldo and Kucuker, 2025), MaxEnt (MAXENT) (Chen et al., 2026b), MaxEnt (MAXNET) (Kang et al., 2025), random forest (RF) (Zhang et al., 2020), surface range envelope (SRE) (Hallgren et al., 2019), and extreme gradient boosting (XGBOOST) (Mu et al., 2025). Because true absences were unavailable, we generated 1, 000 pseudo-absence points using a random strategy. This number provided a stable background sample relative to the spatially thinned presence dataset while maintaining computational efficiency across repeated multi-algorithm calibrations. For each replicate, 75% of occurrence records were used for model calibration and 25% for evaluation, and the split-sample procedure was repeated 10 times. In other words, 10 independent random partitions were generated and each algorithm was fitted 10 times, yielding 120 single-model calibrations before ensemble integration. Model performance was assessed using the area under the ROC curve (AUC) and the true skill statistic (TSS) (Shabani et al., 2016). Predictor importance was quantified using the permutation-based variable-importance procedure implemented in Biomod2; jackknife tests were not used because our analysis focused on cross-algorithm comparability within the ensemble framework. Ensemble forecasts were constructed by retaining only individual models with TSS > 0.75 and aggregating predictions using the committee-averaging approach (EMca) (Wang et al., 2025).

Habitat-suitability rasters were post-processed in ArcGIS 10.8. For cross-scenario comparison, suitability was classified using fixed thresholds rather than scenario-specific natural breaks: unsuitable (0-0.25), marginally suitable (0.25-0.50), and highly suitable (>0.50). The threshold of 0.50 was used to distinguish higher-confidence suitability from transitional habitat and to facilitate consistent comparison across time periods and scenarios; these classes are therefore intended as a comparative framework rather than absolute ecological limits.To quantify spatial redistribution, we calculated the geometric centroid of suitable areas using SDM Toolbox and tracked centroid displacement across scenarios and time periods (Brown, 2014). We also conducted Multivariate Environmental Similarity Surface (MESS) and Most Dissimilar variable (MoD) analyses by comparing each projected grid cell with the environmental ranges represented in the calibration data. In this framework, low or negative MESS values indicate cells with increasingly novel environmental conditions, while MoD identifies the predictor that contributes most strongly to that novelty.

## 3 Results

### 3.1 Model performance

**Figure 3:**
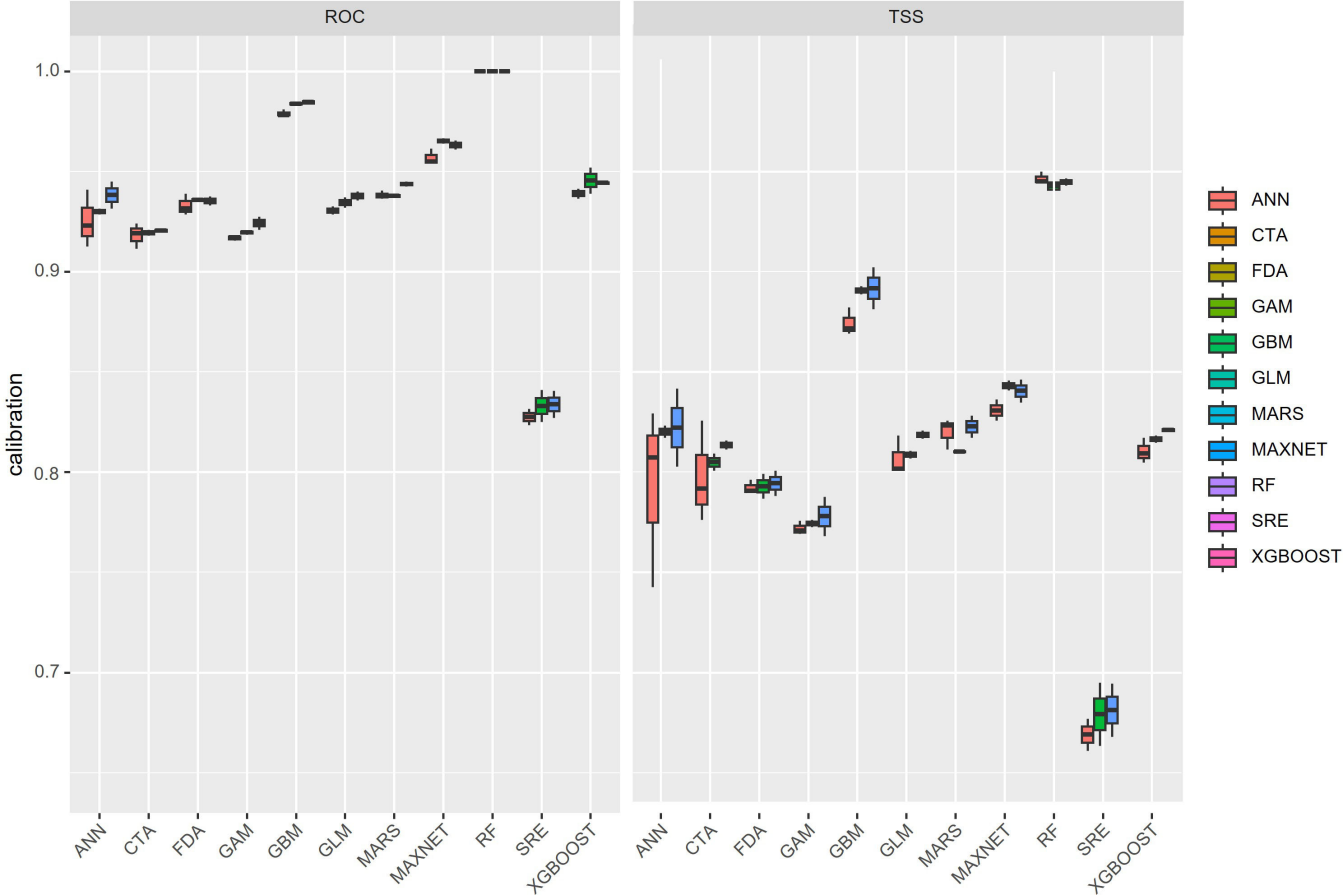
TSS and ROC evaluations of the single models included in the biomod2 workflow.

All single algorithms achieved strong predictive performance (TSS > 0.80 and AUC > 0.90; **Figure 3**). Among the ensemble strategies compared, EMca had the highest overall accuracy (TSS = 0.842; AUC = 0.96; **Table 2**) and was therefore retained for subsequent spatial projections and interpretation.

**Table 2:** Evaluation metrics of the ensemble-modeling strategies tested in this study.

Ensemble model	TSS	ROC
EMmean	0.817	0.947
EMmedian	0.789	0.929
EMca	0.842	0.96
EMwmean	0.819	0.948

TSS, true skill statistic; ROC, receiver operating characteristic. EMmean represents the ensemble mean model; EMmedian represents the ensemble median model; EMca represents the ensemble committee averaging model; EMwmean represents the ensemble weighted mean model.

### 3.2 Environmental drivers of suitability

**Figure 4:**
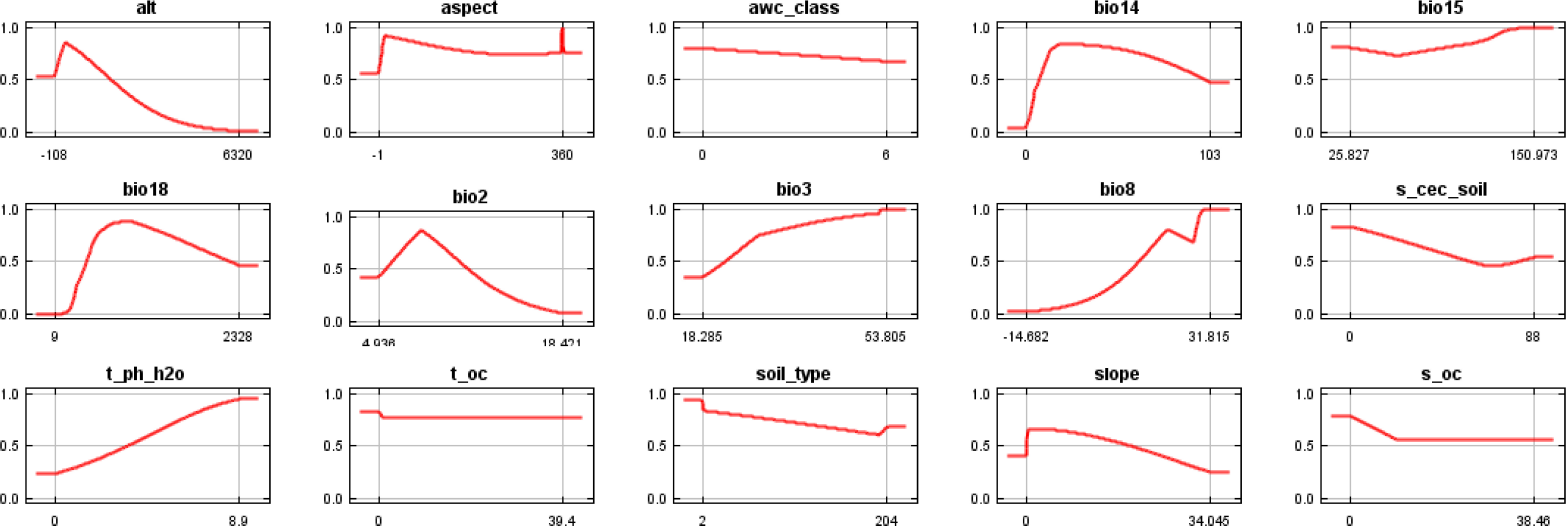
Representative response curves of the major environmental predictors affecting modeled suitability for *B. biternata*.

After collinearity screening, 15 predictors were retained. Permutation-based variable-importance estimates indicated that climatic variables consistently explained the largest share of model variation, whereas topographic and soil variables contributed secondarily. Temperature-related predictors were repeatedly among the most influential, particularly mean diurnal temperature range (bio2), isothermality (bio3), and, depending on the algorithm, mean temperature of the wettest quarter (bio8). Representative response curves for these major predictors are shown in **Figure 4**. For example, GLM/FDA/GAM-type models relied strongly on bio2 and bio3, whereas MAXNET assigned relatively greater importance to bio8, indicating sensitivity to wet-season thermal constraints. Overall, *B. biternata* is associated with warm wet-season conditions, moderate diurnal temperature variability, relatively high isothermality, and non-extreme dry-season precipitation, with edaphic and terrain factors exerting additional but smaller effects at the national scale.

### 3.3 Current potential distribution in China

**Figure 5:**
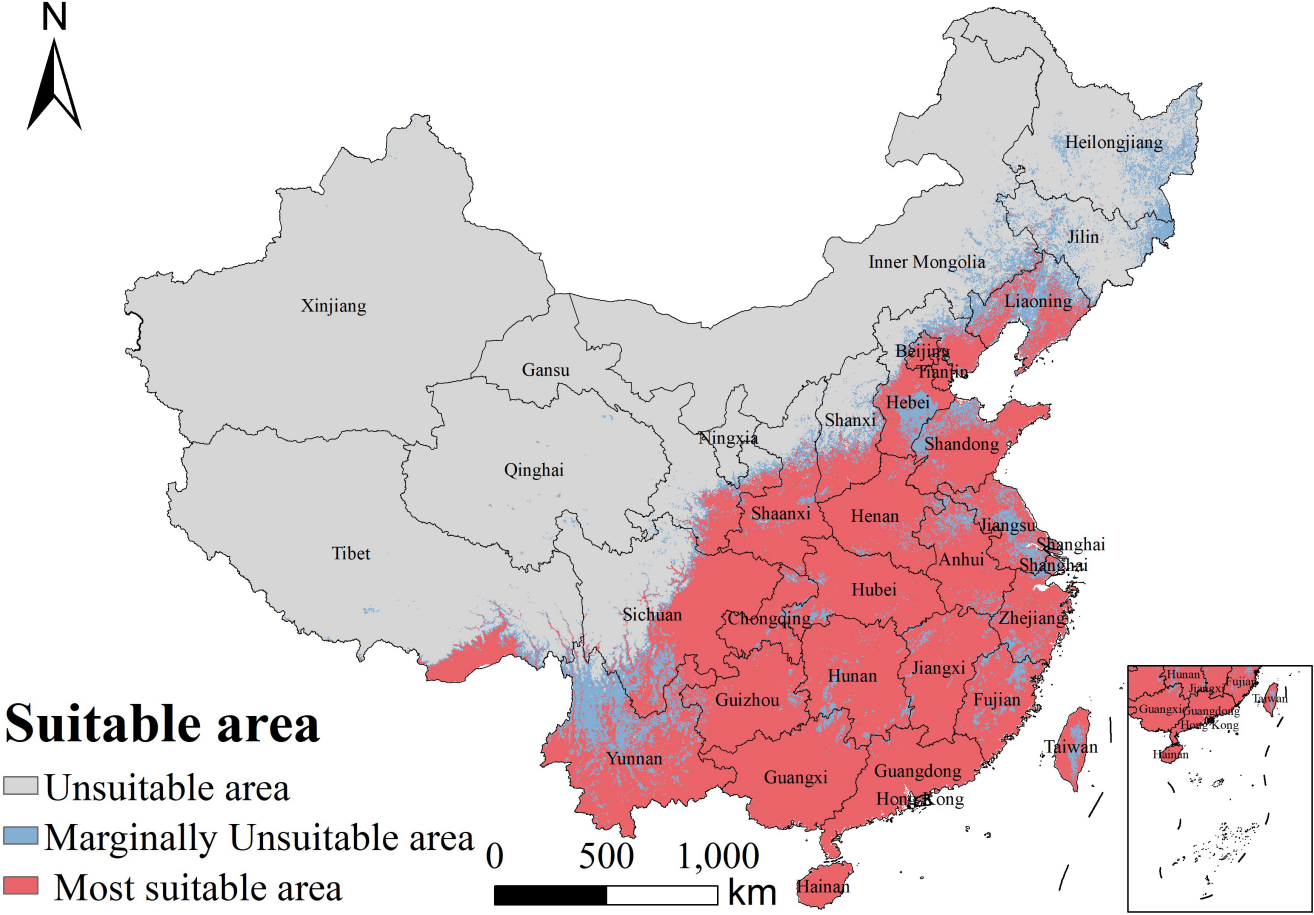
Current potential geographic distribution of *B. biternata* in China, classified as unsuitable, marginally suitable, and highly suitable under the baseline climate.

Using the EMca ensemble, we mapped the potential distribution of *B. biternata* under the current climate baseline. Highly suitable habitat covered 2.68 × 10^5^ km^2^ (17.20%), marginally suitable habitat covered 6.65 × 10^5^ km^2^ (42.68%), and unsuitable habitat covered 6.25 × 10^5^ km^2^ (40.12%). Suitability showed a pronounced southeast–northwest gradient (**Figure 5**). Highly suitable areas (>0.50) formed a broad, largely continuous belt from South China and the southeastern coastal provinces (e.g., Guangdong, Guangxi, Fujian, Zhejiang, including Hainan and Taiwan) through the middle and lower Yangtze River region (e.g., Jiangxi, Hunan, Hubei, Anhui, Jiangsu) and into parts of North China (notably Shandong–Hebei and the Beijing–Tianjin region). In Southwest China, extensive highly suitable habitat was projected across the Yunnan–Guizhou Plateau and the Sichuan Basin. Marginal suitability (0.25–0.50) occurred mainly as transitional zones surrounding the core belt and was more fragmented in Northeast China and along mountain–basin ecotones. Unsuitable habitat (0–0.25) predominated in northwestern arid regions and on the Qinghai–Tibet Plateau, reflecting constraints imposed by cold temperatures, high elevation, and aridity.

### 3.4 Spatiotemporal changes in suitable habitat under future climates

**Figure 6:**
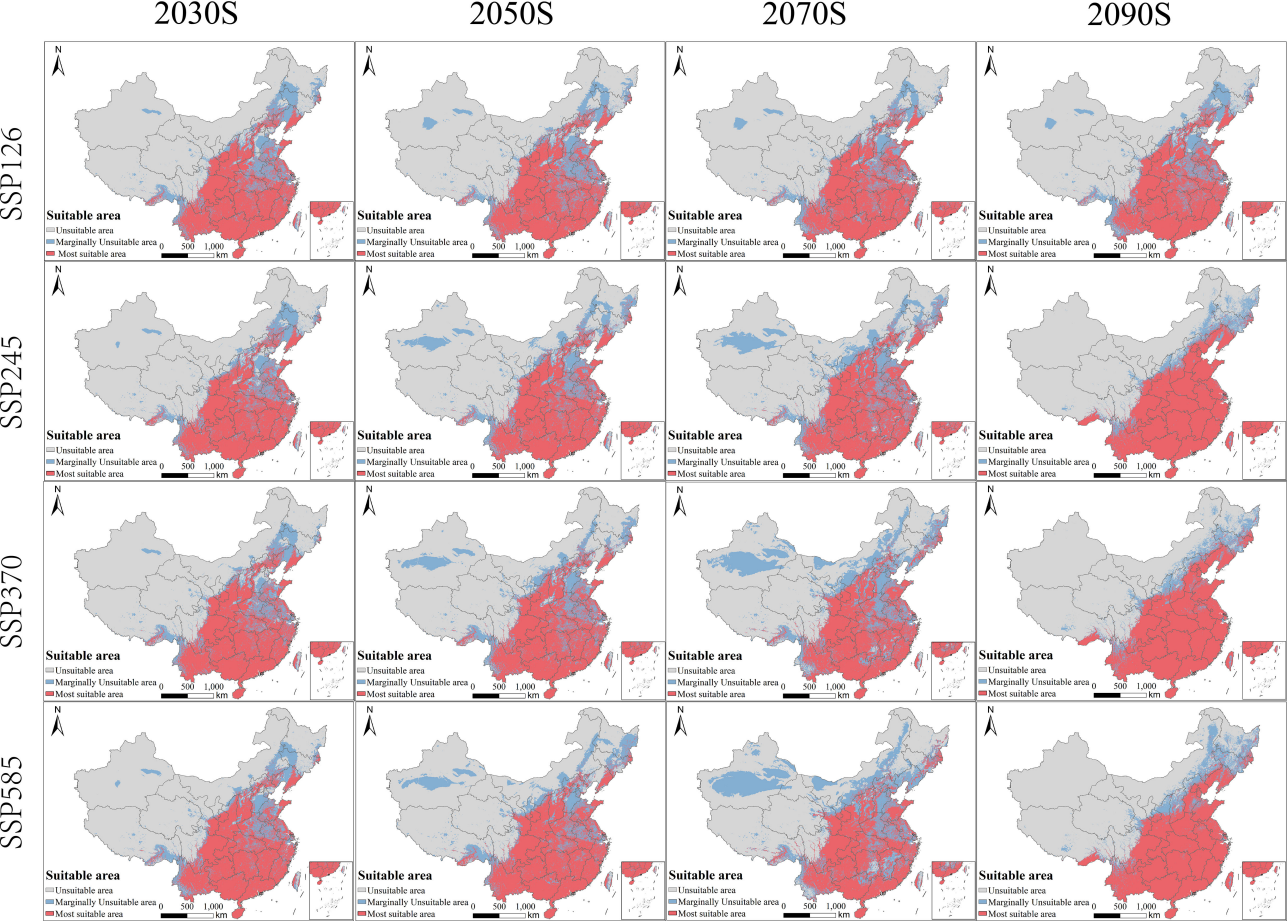
Future potential geographic distribution of *B. biternata* in China under four SSP pathways and four time periods.

Across future SSPs, projected suitable habitat exhibited clear time- and scenario-dependent changes in both composition and spatial pattern (**Table 3**; **Figure 6**). Here, SSP126, SSP245, SSP370, and SSP585 represent low-, intermediate-, medium-high-, and high-forcing socioeconomic-climate pathways, respectively. The future periods analyzed were 2021–2040, 2041–2060, 2061–2080, and 2081–2100, which are discussed below as the 2030s, 2050s, 2070s, and 2090s for interpretive convenience. Relative to the baseline, the proportion of highly suitable habitat generally increased during 2021-2080, typically reaching ~25-28% across SSPs (SSP126: 25.52-26.35%; SSP245: 25.94-27.40%; SSP370: 25.83-26.69%; SSP585: 25.31-28.44%). Over the same periods, marginally suitable habitat declined to ~12–25%, whereas unsuitable habitat remained relatively high (~50–62%). These trajectories indicate that near- to mid-century gains were driven mainly by upgrading within the existing suitability belt (marginal to high), rather than by widespread conversion of currently unsuitable regions.

**Table 3:** This pattern suggests that stronger late-century forcing may yield broader transitional belts that are permissive for occurrence but less consistently optimal for persistent populations or high-quality medicinal production.

Climate scenarios	Time period	Unsuitable area (×10^5^ km^2^)	Unsuitable area (%)	Marginally unsuitable area (×10^5^ km^2^)	Marginally suitable area (%)	Highly suitable area (×10^5^ km^2^)	Highly suitable area (%)
Current	1970-2000	6.25	40.12	6.65	42.68	2.68	17.20
SSP126	2021-2040	5.88	61.19	1.26	13.11	2.47	25.70
	2041-2060	5.80	60.42	1.35	14.06	2.45	25.52
	2061-2080	5.89	61.35	1.24	12.92	2.47	25.73
	2081-2100	5.78	60.21	1.29	13.44	2.53	26.35
SSP245	2021-2040	5.94	61.88	1.17	12.19	2.49	25.94
	2041-2060	5.50	57.29	1.47	15.31	2.63	27.40
	2061-2080	5.26	54.79	1.73	18.02	2.61	27.19
	2081-2100	5.79	35.96	7.22	44.84	3.09	19.19
SSP370	2021-2040	5.87	61.21	1.16	12.10	2.56	26.69
	2041-2060	5.58	58.19	1.48	15.43	2.53	26.38
	2061-2080	4.95	51.56	2.17	22.60	2.48	25.83
	2081-2100	5.70	33.83	8.05	47.77	3.10	18.40
SSP585	2021-2040	5.81	60.52	1.23	12.81	2.56	26.67
	2041-2060	5.32	55.42	1.55	16.15	2.73	28.44
	2061-2080	4.80	50.00	2.37	24.69	2.43	25.31
	2081-2100	5.55	30.25	9.72	52.97	3.08	16.78

By late century (2081–2100), trajectories diverged among SSPs. Under SSP126, habitat composition remained comparatively stable, with modest fluctuations and a persistently high unsuitable fraction (~60%). In contrast, under SSP245/SSP370/SSP585, suitability shifted toward marginal conditions: marginally suitable area expanded markedly (44.84–52.97%), while the highly suitable fraction declined (~16.78–19.19%). This pattern suggests that stronger late-century forcing may yield broader transitional belts that are permissive for occurrence but less consistently optimal for persistent populations or high-quality medicinal production.

Spatially, projections indicated continued expansion along the northern and northeastern range margins across scenarios and periods, whereas contractions were comparatively limited and patchy. Toward late century—especially under higher forcing—expansion was increasingly accompanied by localized contractions and stronger class transitions, consistent with the late-century increase in marginal suitability (**Table 3**). This northward and northeastward shift is likely linked to warming-induced relaxation of low-temperature constraints and lengthening of the thermal growing season at higher latitudes, while monsoonal moisture still supports establishment in newly suitable transition zones.

**Figure 7:**
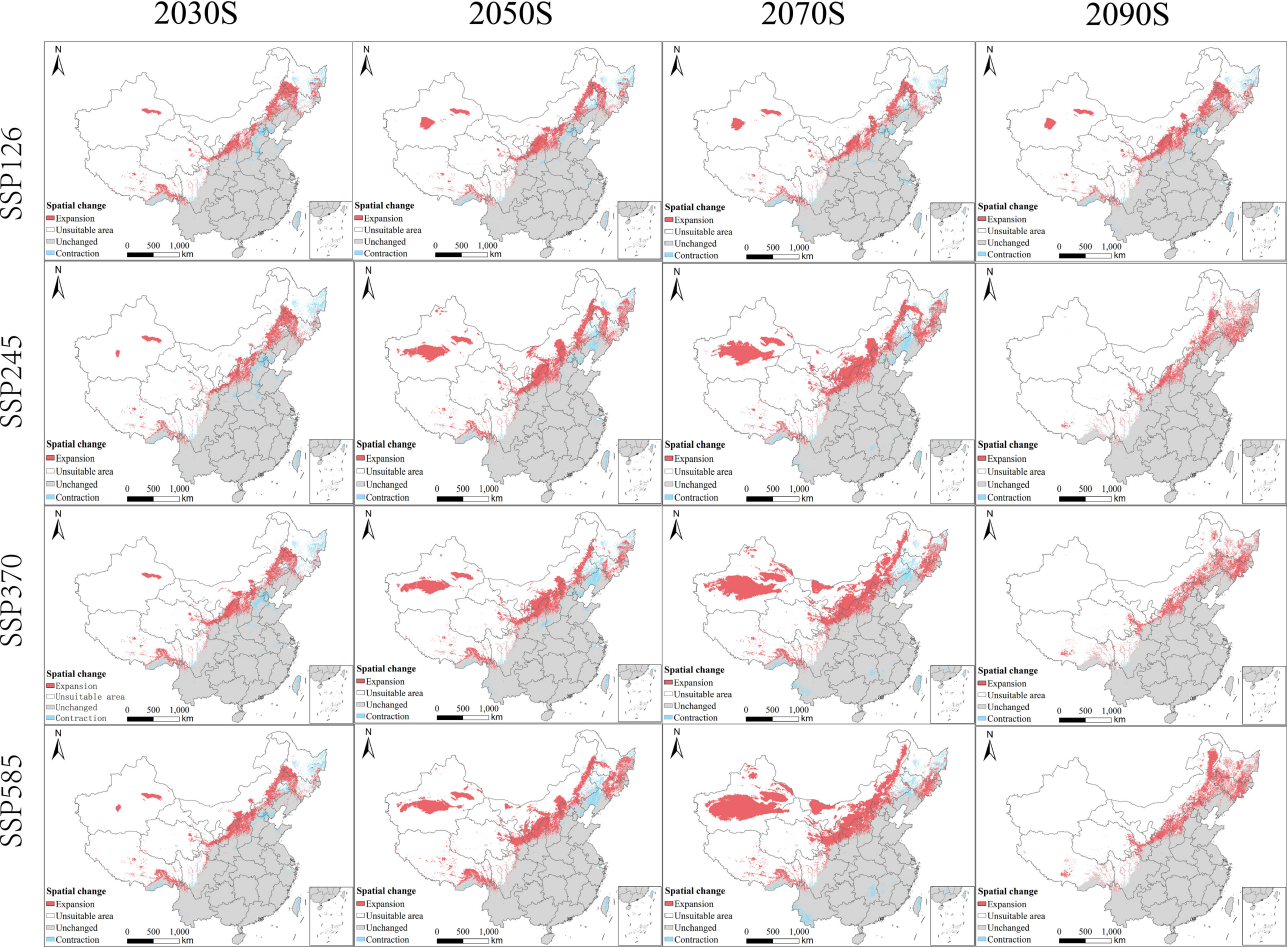
Comparison of current and future proportions of unsuitable, marginally suitable, and highly suitable habitat for *B. biternata* under different climate sce.

To complement the quantitative analysis of range shifts, we spatially compared future suitable habitats with the current baseline distribution of *Bidens biternata* (**Figure 7**) Across all scenarios, a consistent and pronounced northward range shift was observed, characterized by heterogeneous patterns of expansion, contraction, and stability.

### 3.5 Multivariate environmental similarity surface and most dissimilar variables

**Figure 8:**
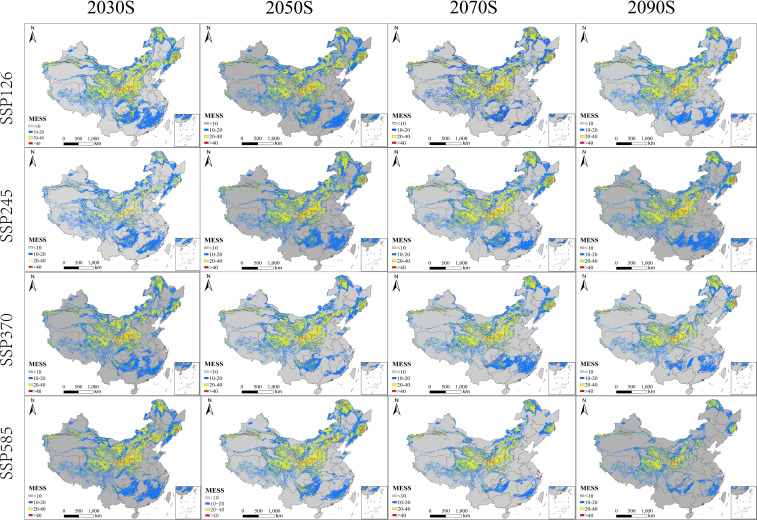
Multivariate environmental similarity surface (MESS) maps for *B. biternata* under different future climate scenarios.

MESS maps indicated that most projections were made within moderate-to-high environmental similarity space, while also revealing geographically structured regions of climatic novelty (**Figure 8**). Areas of higher similarity (MESS 20–40) formed a persistent belt across central–eastern China, broadly corresponding to the North China Plain–middle/lower Yangtze–Sichuan Basin corridor. Very high similarity (MESS > 40) occurred as localized hotspots, mainly in eastern monsoonal regions, and was most evident during mid-century periods under SSP126–SSP245. In contrast, low similarity (MESS < 10), implying greater extrapolation risk, was consistently concentrated in western China (e.g., the Qinghai–Tibet Plateau and parts of the northwest) and became more prominent toward late century, particularly under SSP370–SSP585, with additional patches near projected expansion fronts.

**Figure 9:**
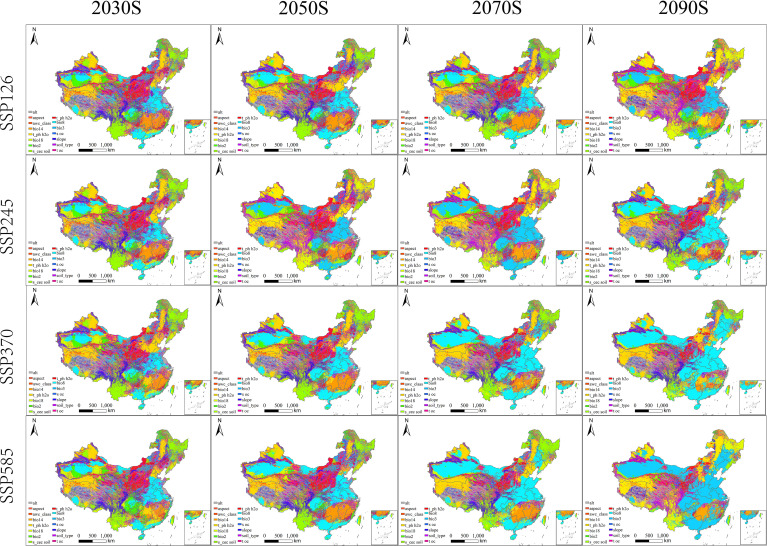
Most dissimilar variable (MoD) maps for *B. biternata* under different future climate scenarios.

MoD maps showed that the dominant sources of environmental dissimilarity varied spatially but followed consistent tendencies across scenarios and periods (**Figure 9**). Along the northern and northeastern frontiers—where MESS values were often low—temperature-related predictors (e.g., bio2, bio3, bio8) and, in some regions, precipitation variables (e.g., bio14, bio18) most frequently became the first variables to fall outside the ranges represented in the calibration data. Within the core suitability belt where MESS values were generally higher, MoD patterns were more heterogeneous and often shifted toward edaphic or topographic variables (e.g., soil pH, soil type, soil carbon/CEC, slope, and elevation), reflecting finer-scale environmental contrasts. Notably, under SSP370–SSP585 in the late century, climatic variables increasingly dominated MoD in northern expansion zones, consistent with the concurrent expansion of low-similarity MESS classes. Because MoD identifies the single variable contributing most strongly to climatic novelty for each period-specific projection, the dominant variable can change through time within the same SSP as climate trajectories alter which predictor first exceeds the training envelope.

### 3.6 Centroid shifts of suitable habitat under climate change

**Figure 10:**
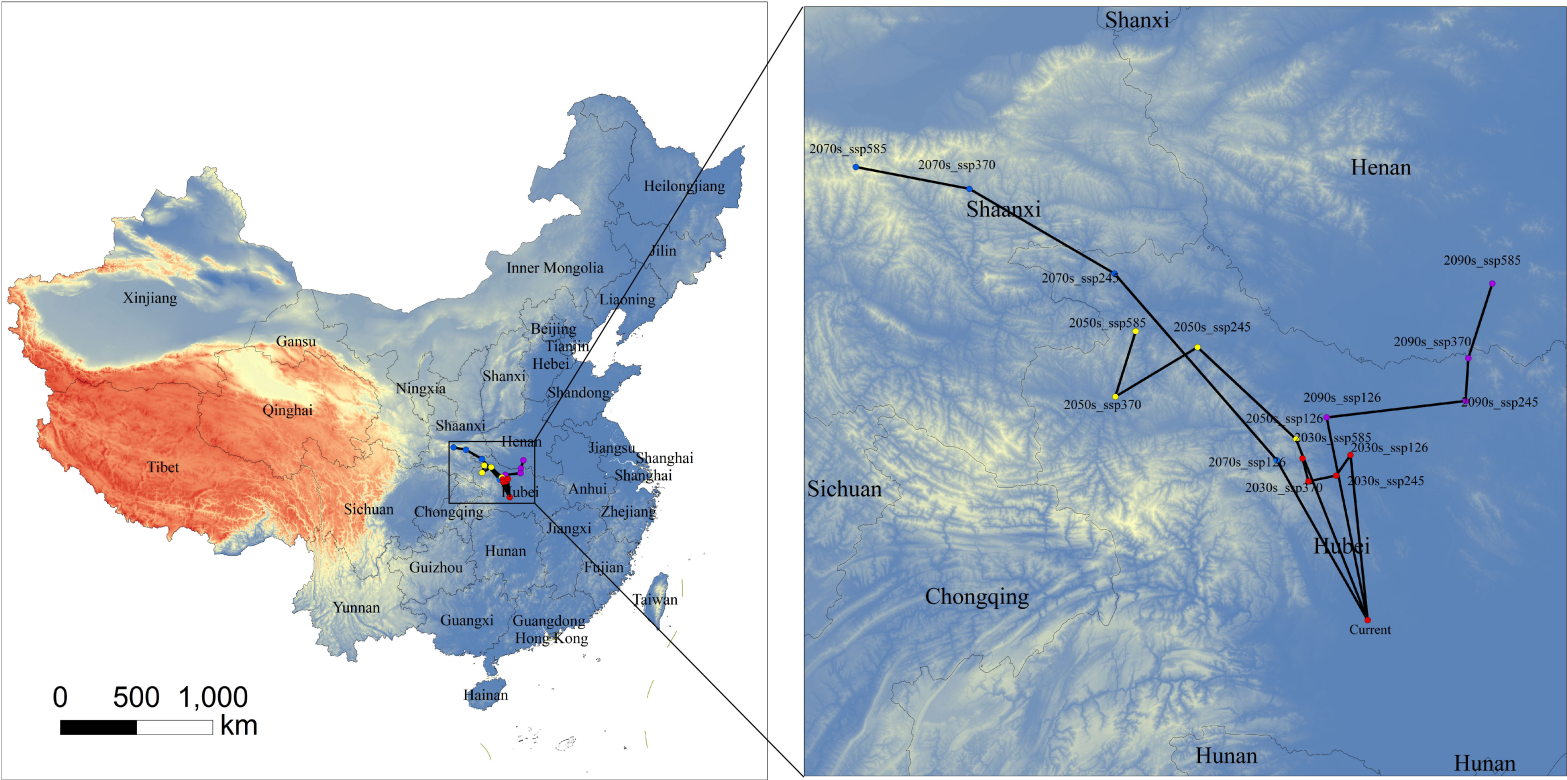
Centroid migration of the suitable-habitat core of *B. biternata* under different future climate change scenarios.

Centroid analysis revealed clear scenario- and time-dependent shifts in the geographic center of suitable habitat (**Figure 10**). Overall, centroid trajectories indicated a consistent northward to northwestward displacement relative to the current position, implying a systematic poleward redistribution of suitability.

In the near term (2030s), centroid shifts were small across scenarios. By mid-century (2050s), movements became more pronounced and began to diverge among SSPs, with larger displacements generally occurring under higher forcing. The greatest displacement occurred in the 2070s, particularly under SSP370 and SSP585, with the centroid moving northwestward toward the Shaanxi–Shanxi transition zone.

By late century (2090s), trajectories diverged further. Under SSP126, the centroid remained relatively close to mid-century positions, suggesting a more stable spatial configuration. In contrast, under SSP245–SSP585, the centroid continued to shift away from the current location, with displacement increasing with radiative forcing, reflecting continued redistribution along range margins.

## 4 Discussion

### 4.1 Climatic controls on suitability

Across algorithms, climatic predictors explained most of the variation in habitat suitability, whereas topographic and edaphic factors contributed secondarily (Gonçalves et al., 2016). Temperature-related variables—especially mean diurnal temperature range (bio2) and isothermality (bio3), and in some models mean temperature of the wettest quarter (bio8)—were consistently influential. Ecologically, this suggests that *B. biternata* is constrained not only by overall warmth, but also by the stability of thermal conditions during the growing season and by the balance between day–night and annual temperature variation. For an annual medicinal herb, such thermal regulation can influence germination timing, vegetative growth, flowering, and seed set. Moisture-related variables, including precipitation of the driest month and precipitation of the warmest quarter, likely further modulate successful establishment by shaping seasonal water availability. Climatic variables also outperform soil and topographic variables at the national scale because climate provides the broad-scale energy and water template that structures range limits, whereas soil and terrain more often refine habitat quality locally or regionally. Together, these results indicate that *B. biternata* is best adapted to warm monsoonal environments with relatively stable wet-season thermal conditions, whereas soil and terrain mainly refine suitability at subregional scales rather than control the national-scale distribution pattern (Xu et al., 2018).

### 4.2 Current suitability pattern and implications for management

Under the current climate baseline, suitability exhibits a pronounced southeast–northwest gradient. Highly suitable habitat forms a broad belt across South China, the middle–lower Yangtze region, parts of North China, and warm–humid basins and lowlands in Southwest China, whereas unsuitable habitat is concentrated in northwestern arid regions and on the Qinghai–Tibet Plateau (**Figure 5**). Quantitatively, the model estimates 2.68 × 10^5 km^2 of highly suitable habitat (17.20%), 6.65 × 10^5 km^2 of marginally suitable habitat (42.68%), and 6.25 × 10^5 km^2 of unsuitable habitat (40.12%) (**Table 3**). From a medicinal-resource perspective, these patterns suggest that wild harvesting and semi-cultivation are most feasible in monsoonal eastern and southern China, whereas expansion into arid northwestern or high-elevation regions is unlikely without substantial mitigation of climatic constraints (Papageorgiou et al., 2020; Treurnicht et al., 2021).

### 4.3 Future redistribution of suitable habitat under climate change

Projected changes in suitability were strongly scenario- and time-dependent (Anderson et al., 2012; Ferreira et al., 2016; DeWeber and Wagner, 2018). During 2021–2080, the proportion of highly suitable habitat increased to ~25–28% across SSPs, while marginal suitability declined and unsuitable area remained relatively high. These near- to mid-term gains appear to arise mainly from upgrading within the current suitability belt (marginal to high) rather than from large-scale conversion of presently unsuitable regions. In practical terms, this suggests that climate warming may initially enhance the continuity of the existing core distribution zone before stronger late-century forcing generates broader but less optimal transitional belts.

By late century (2081–2100), pathways diverged. Under SSP126, habitat composition remained comparatively stable and the unsuitable fraction stayed high (~60%). Under SSP245, SSP370, and SSP585, suitability shifted toward marginal conditions, with marginally suitable habitat expanding to 44.84–52.97% and the highly suitable fraction declining to ~16.78–19.19%. This implies that stronger late-century forcing may produce broader transition zones that are climatically permissive for occurrence but less consistently optimal for stable population persistence or high-quality medicinal production (McMichael et al., 2015; Panda, 2018). Therefore, an increase in climatically acceptable area should not automatically be interpreted as an equivalent increase in reliable cultivation land or medicinal resource quality.

Spatially, suitability projections indicate continued expansion along northern and northeastern margins, with increasingly mixed signals of expansion and contraction toward late century under higher forcing. Management should therefore prioritize (i) identifying and safeguarding stable high-suitability areas that are likely to persist as near-term refugia and production cores (Gao et al., 2025), and (ii) evaluating emerging suitable margins in northern and northeastern China, where establishment may become feasible but interannual suitability, projection uncertainty, and climatic novelty may all be higher (Solarik et al., 2018).

### 4.4 Centroid migration and planning implications

Centroid trajectories support an overall northward to northwestward displacement of the suitable-habitat core, with modest shifts in the 2030s, stronger divergence by the 2050s, and the greatest displacement in the 2070s under higher forcing. These trends suggest that cultivation zoning and germplasm planning may need to progressively expand toward higher latitudes and climatically suitable transition zones, while conservation and resource-management efforts should also safeguard the current high-suitability belt that is likely to remain a persistence core under most scenarios (Bradley et al., 2012; Aparecido et al., 2024).

### 4.5 Climate refugia and implications for sustainable utilization

The results suggest several areas that may function as climatic refugia for *B. biternata*. In this study, refugia are inferred from the overlap among current high suitability, relatively stable future suitability, limited centroid departure, and moderate-to-high MESS similarity. On this basis, the middle-lower Yangtze region, parts of South China, the Sichuan Basin, and sections of the Yunnan-Guizhou Plateau appear to be the most plausible persistence areas. These regions should be prioritized for *in situ* conservation, germplasm preservation, and long-term population monitoring because they are more likely to maintain suitable hydrothermal conditions across multiple scenarios.

The distribution patterns revealed here can also guide sustainable utilization. In persistent core areas, management should emphasize regulated wild harvesting, semi-cultivation, provenance selection, and the establishment of seed or germplasm reserves to reduce pressure on natural populations. In contrast, newly emerging suitable areas in northern and northeastern China should be treated as trial-introduction zones rather than immediate large-scale production bases, because climatic novelty is higher there and model transferability is less certain. A phased strategy combining field validation, pilot cultivation, quality assessment of medicinal materials, and adaptive agronomic management would better align conservation with future utilization.

## 5 Conclusions

Using an EMca ensemble SDM framework implemented in Biomod2, together with spatially filtered occurrences records and a low-collinearity predictor set, we produced robust projections of the potential distribution of *B.biternata* in China (TSS = 0.842; AUC = 0.96). Under the current climate baseline, suitable habitat is concentrated in monsoonal eastern and southern China and parts of Southwest China, generating a clear southeast–northwest gradient.Climatic predictors—particularly temperature variability and wet-season thermal conditions (bio2, bio3, bio8)—dominate habitat suitability, whereas topography and soil contribute secondarily.

Future projections indicate an early- to mid-century increase in highly suitable habitat (~25–28% during 2021–2080) across SSPs, followed by late-century divergence under higher forcing (SSP245/SSP370/SSP585), with broader marginal-suitability belts and reduced highly suitable fractions. Suitability expands mainly along northern and northeastern margins, and the habitat centroid shifts predominantly northward to northwestward. MESS/MoD diagnostics further show increasing climatic novelty at expansion fronts, suggesting that stable suitability cores in the middle-lower Yangtze region, South China, the Sichuan Basin, and parts of Southwest China are likely to be more reliable targets for conservation and cultivation planning than climatically novel frontier zones. By integrating ensemble modeling, centroid tracking, and climatic-novelty diagnostics, this study provides a decision-oriented framework for distinguishing persistent medicinal-plant cores from uncertain expansion areas under climate change.

This study has several limitations. First, although occurrence records were spatially filtered, residual sampling bias and database uncertainty may still influence model calibration. Second, future projections were based on a single general circulation model (BCC-CSM2-MR), so inter-model climate uncertainty was not fully captured. Third, the ensemble SDM did not explicitly incorporate dispersal limitation, land-use change, biotic interactions, or variation in medicinal quality traits, all of which may affect the realized future distribution and utilization value of *B. biternata*. Future work should therefore integrate multi-GCM ensembles, field validation in expansion zones, demographic and dispersal processes, and quality-oriented assessments to improve conservation and cultivation recommendations.

## Data Availability

The raw data supporting the conclusions of this article will be made available by the authors, without undue reservation.

## References

[B1] Anderson A. S. Reside A. E. Vanderwal J. J. Shoo L. P. Pearson R. G. Williams S. E. (2012). Immigrants and refugees: the importance of dispersal in mediating biotic attrition under climate change. Global Change Biol. 18, 2126–2134. doi: 10.1111/j.1365-2486.2012.02683.x. PMID: 40046247

[B2] Aparecido L. E. D. Torsoni G. B. de Lima R. F. Baratti A. C. C. Rossi M. F. D. dos Santos A. F. . (2024). Climate zoning: identifying suitable regions for the occurrence of Alternaria Brown spot in tangerine trees in Brazil. Pest. Manage. Sci. 80, 1615–1631. doi: 10.1002/ps.7894. PMID: 37985580

[B3] Babu K. N. Gour R. Ayushi K. Ayyappan N. Parthasarathy N. (2023). Environmental drivers and spatial prediction of forest fires in the Western Ghats biodiversity hotspot, India: An ensemble machine learning approach. For. Ecol. Manage. 540, 121057. doi: 10.1016/j.foreco.2023.121057. PMID: 38826717

[B4] Bradley B. A. Estes L. D. Hole D. G. Holness S. Oppenheimer M. Turner W. R. . (2012). Predicting how adaptation to climate change could affect ecological conservation: secondary impacts of shifting agricultural suitability. Divers. Distrib. 18, 425–437. doi: 10.1111/j.1472-4642.2011.00875.x. PMID: 40046247

[B5] Brown J. L. (2014). SDMtoolbox: a python-based GIS toolkit for landscape genetic, biogeographic and species distribution model analyses. Methods Ecol. Evol. 5, 694–700. doi: 10.1111/2041-210x.12200. PMID: 29230356 PMC5721907

[B6] Chen I. C. Hill J. K. Ohlemüller R. Roy D. B. Thomas C. D. (2011). Rapid range shifts of species associated with high levels of climate warming. Science 333, 1024–1026. doi: 10.1126/science.1206432. PMID: 21852500

[B7] Chen R. Luo F. M. Yao W. H. Yang R. M. Huang L. Li H. . (2026a). Ecological niche differentiation and distribution dynamics revealing climate change responses in the Chinese genus. Plants-Basel 15, 162. doi: 10.3390/plants15010162. PMID: 41515108 PMC12788128

[B8] Chen Z. L. Zhang Y. Li M. S. (2026b). Evaluating the performance of species distribution models Biomod2 and MaxEnt in forest grassland fire prediction: an example from Sichuan Province, China. Geomatics Nat. Hazards Risk 17, 2612203. doi: 10.1080/19475705.2025.2612203. PMID: 37339054

[B9] China, E.C.o.F.o (2007). Flora of China (Beijing: Sci. Press).

[B10] Das A. C. Noguchi R. Ahamed T. (2020). Integrating an expert system, GIS, and satellite remote sensing to evaluate land suitability for sustainable tea production in Bangladesh. Remote Sens. 12, 4136. doi: 10.3390/rs12244136. PMID: 30654563

[B11] DeWeber J. T. Wagner T. (2018). Probabilistic measures of climate change vulnerability, adaptation action benefits, and related uncertainty from maximum temperature metric selection. Global Change Biol. 24, 2735–2748. doi: 10.1111/gcb.14101. PMID: 29468779

[B12] Di Cola V. Broennimann O. Petitpierre B. Breiner F. T. D'Amen M. Randin C. . (2017). ecospat: an R package to support spatial analyses and modeling of species niches and distributions. Ecography 40, 774–787. doi: 10.1111/ecog.02671. PMID: 40046247

[B13] Dieleman C. M. Branfireun B. A. Mclaughlin J. W. Lindo Z. (2015). Climate change drives a shift in peatland ecosystem plant community: implications for ecosystem function and stability. Global Change Biol. 21, 388–395. doi: 10.1111/gcb.12643. PMID: 24957384

[B14] Farsi M. Kalantar M. Zeinalabedini M. Vazifeshenas M. R. (2023). First assessment of Iranian pomegranate germplasm using targeted metabolites and morphological traits to develop the core collection and modeling of the current and future spatial distribution under climate change conditions. PloS One 18, e0265977. doi: 10.1371/journal.pone.0265977. PMID: 36735649 PMC9897574

[B15] Feng G. Xiong Y. J. Wei H. Y. Li Y. Mao L. F. (2023). Endemic medicinal plant distribution correlated with stable climate, precipitation, and cultural diversity. Plant Divers. 45, 479–484. doi: 10.1016/j.pld.2022.09.007. PMID: 37601541 PMC10435908

[B16] Feng X. Peterson A. T. Aguirre-Lopez L. J. Burger J. R. Chen X. Papes M. (2024). Rethinking ecological niches and geographic distributions in face of pervasive human influence in the Anthropocene. Biol. Rev. 99, 1481–1503. doi: 10.1111/brv.13077. PMID: 38597328

[B17] Ferreira M. T. Cardoso P. Borges P. A. Gabriel R. de Azevedo E. B. Reis F. . (2016). Effects of climate change on the distribution of indigenous species in oceanic islands (Azores). Clim. Change 138, 603–615. doi: 10.1007/s10584-016-1754-6. PMID: 30311153

[B18] Fick S. E. Hijmans R. J. (2017). WorldClim 2: new 1-km spatial resolution climate surfaces for global land areas. Int. J. Climatol. 37, 4302–4315. doi: 10.1002/joc.5086. PMID: 41531421

[B19] Gan T. He Z. Xu D. Chen J. Zhang H. Wei X. . (2025). Modeling the potential distribution of Hippophae rhamnoides in China under current and future climate scenarios using the biomod2 model. Front. Plant Sci. 16, 1533251. doi: 10.3389/fpls.2025.1533251. PMID: 40256597 PMC12006153

[B20] Gao K. M. Wang C. M. Wu H. Y. (2025). Safeguarding a national treasure: modeling the climate-driven habitat shift of the national key protected in China. Ind. Crops Prod. 235, 121789. doi: 10.1016/j.indcrop.2025.121789. PMID: 38826717

[B21] Giraldo D. C. Kucuker D. M. (2025). Comparative analysis of Biomod2 statistical and machine learning methods for Lactarius deliciosus distribution in Refahiye, Turkiye. Fungal Biol. 129, 101638. doi: 10.1016/j.funbio.2025.101638. PMID: 40915676

[B22] Gonçalves J. Alves P. Pôças I. Marcos B. Sousa-Silva R. Lomba A. . (2016). Exploring the spatiotemporal dynamics of habitat suitability to improve conservation management of a vulnerable plant species. Biodivers. Conserv. 25, 2867–2888. doi: 10.1007/s10531-016-1206-7. PMID: 30311153

[B23] Gray J. M. Humphreys G. S. Deckers J. A. (2011). Distribution patterns of World Reference Base soil groups relative to soil forming factors. Geoderma 160, 373–384. doi: 10.1016/j.geoderma.2010.10.006. PMID: 38826717

[B24] Gu R. Wei S. P. Li J. R. Zheng S. H. Li Z. T. Liu G. L. . (2024). Predicting the impacts of climate change on the geographic distribution of moso bamboo in China based on biomod2 model. Eur. J. For. Res. 143, 1499–1512. doi: 10.1007/s10342-024-01706-9. PMID: 30311153

[B25] Guan Y. L. Liu J. G. Cui W. H. Chen D. L. Zhang J. K. Lu H. W. . (2024). Elevation regulates the response of climate heterogeneity to climate change. Geophys. Res. Lett. 51, e2024GL109483. doi: 10.1029/2024GL109483. PMID: 29143083

[B26] Guo J. Q. Zhou Z. H. Feng T. Liu J. C. Liu J. Zeng X. M. (2025). Prediction of potential suitable areas for dominant tree species in China under future climate change scenarios. J. Plant Ecol. 18, rtaf103. doi: 10.1093/jpe/rtaf103

[B27] Hallgren W. Santana F. Low-Choy S. Zhao Y. Mackey B. (2019). Species distribution models can be highly sensitive to algorithm configuration. Ecol. Modell. 408, 108719. doi: 10.1016/j.ecolmodel.2019.108719. PMID: 38826717

[B28] Hao T. X. Elith J. Lahoz-Monfort J. J. Guillera-Arroita G. (2020). Testing whether ensemble modelling is advantageous for maximising predictive performance of species distribution models. Ecography 43, 549–560. doi: 10.1111/ecog.04890. PMID: 40046247

[B29] Kang Y. Lin F. Yin J. Han Y. Zhu M. Guo Y. . (2025). Projected distribution patterns of Alpinia officinarum in China under future climate scenarios: insights from optimized Maxent and Biomod2 models. Front. Plant Sci. 16, 1517060. doi: 10.3389/fpls.2025.1517060. PMID: 40017818 PMC11866951

[B30] Kaviani S. Sohn I. (2021). Application of complex systems topologies in artificial neural networks optimization: an overview. Expert Syst. Appl. 180, 115073. doi: 10.1016/j.eswa.2021.115073. PMID: 38826717

[B31] Kinuthia D. G. Muriithi A. W. Mwangi P. W. (2016). Freeze dried extracts of Bidens biternata (Lour.) Merr. and Sheriff. show significant antidiarrheal activity in in-vivo models of diarrhea. J. Ethnopharmacol. 193, 416–422. doi: 10.1016/j.jep.2016.09.041. PMID: 27664442

[B32] Liu Y. W. Zhao L. Tan G. R. Shen X. Y. Nie S. P. Li Q. Q. . (2022). Evaluation of multidimensional simulations of summer air temperature in China from CMIP5 to CMIP6 by the BCC models: from trends to modes. Adv. Clim. Change Res. 13, 28–41. doi: 10.1016/j.accre.2021.12.001. PMID: 38826717

[B33] Liu Z. Q. Zhou L. Y. Yan B. Y. Liu Z. G. (2025). Comprehensive assessment of climate-driven distribution dynamics and anthocyanin variation in using MaxEnt, HPLC, and Chemometric approaches. Ind. Crops Prod. 233, 121359. doi: 10.1016/j.indcrop.2025.121359. PMID: 38826717

[B34] Ma X. H. Guo X. D. Xi S. Y. Wang Y. Q. Wu G. T. Jin L. (2025). Biomod2 modeling for predicting the potential ecological distribution of Lilium Davidii var. Willmottiae (E. H. Wilson) Raffill. Sci. Rep. 15, 34346. doi: 10.1038/s41598-025-16837-1. PMID: 41038880 PMC12491542

[B35] Manukyan A. (2019). Secondary metabolites and their antioxidant capacity of Caucasian endemic thyme (Ronn.) as affected by environmental stress. J. Appl. Res. Med. Aromat. Plants 13, 100209. doi: 10.1016/j.jarmap.2019.100209. PMID: 38826717

[B36] McMichael A. J. Butler C. D. Dixon J. (2015). Climate change, food systems and population health risks in their eco-social context. Public Health 129, 1361–1368. doi: 10.1016/j.puhe.2014.11.013. PMID: 25896548

[B37] Mu K. L. Ran F. Feng Y. Q. Guo C. M. Luo Q. M. Wang T. J. . (2025). Integrated evaluation of habitat suitability and spatial prediction of medicinal quality for using species distribution models and machine learning approaches. Ind. Crops Prod. 236, 136751. doi: 10.1016/j.indcrop.2025.121937. PMID: 38826717

[B38] Naughtin S. R. Castilla A. R. Smith A. B. Strand A. E. Dawson A. Hoban S. . (2025). Integrating genomic data and simulations to evaluate alternative species distribution models and improve predictions of glacial refugia and future responses to climate change. Ecography 2025, e07196. doi: 10.1111/ecog.07196. PMID: 40046247

[B39] Oeser J. Zurell D. Mayer F. Çoraman E. Toshkova N. Deleva S. . (2024). The best of two worlds: using stacked generalisation for integrating expert range maps in species distribution models. Global Ecol. Biogeogr. 33, e13911. doi: 10.1111/geb.13911. PMID: 40046247

[B40] Panda A. (2018). Transformational adaptation of agricultural systems to climate change. Wiley Interdiscip. Reviews-Climate Change 9, e520. doi: 10.1002/wcc.520. PMID: 41531421

[B41] Papageorgiou D. Bebeli P. J. Panitsa M. Schunko C. (2020). Local knowledge about sustainable harvesting and availability of wild medicinal plant species in Lemnos island, Greece. J. Ethnobiol. Ethnomed. 16, 36. doi: 10.1186/s13002-020-00390-4. PMID: 32560660 PMC7304145

[B42] Pizarro S. Pricope N. G. Vera J. Cruz J. Lastra S. Solórzano-Acosta R. . (2025). Comprehensive spatial mapping of metals and metalloids in the Peruvian Mantaro Valley using advanced geospatial data integration. Geoderma 453, 117138. doi: 10.1016/j.geoderma.2024.117138. PMID: 38826717

[B43] Safaei M. Rezayan H. Firouzabadi P. Z. Sadidi J. (2021). Optimization of species distribution models using a genetic algorithm for simulating climate change effects on Zagros forests in Iran. Ecol. Inf. 63, 101288. doi: 10.1016/j.ecoinf.2021.101288. PMID: 38826717

[B44] Serafini C. Cosentino F. Amori G. Maiorano L. (2025). Modelling species distribution at the boundaries of the Earth's climate. Global Ecol. Biogeogr. 34, e70082. doi: 10.1111/geb.70082. PMID: 40046247

[B45] Shabani F. Kumar L. Ahmadi M. (2016). A comparison of absolute performance of different correlative and mechanistic species distribution models in an independent area. Ecol. Evol. 6, 5973–5986. doi: 10.1002/ece3.2332. PMID: 27547370 PMC4983607

[B46] Shan Z. J. Zhang Q. Peng D. X. Ye J. F. Cao L. Chen Z. D. . (2023). Assessing conservation priorities of threatened medicinal plants in China: a new comprehensive phylogenetic scoring system. J. Syst. Evol. 61, 709–718. doi: 10.1111/jse.12903. PMID: 40046247

[B47] Soilhi Z. Hafsi C. Mekki M. (2024). Ensemble modeling to predict current and future distribution of Ailanthus altissima (Mill.) Swingle in Tunisia. Biol. Invasions 27, 53. doi: 10.1007/s10530-024-03523-y. PMID: 30311153

[B48] Solarik K. A. Messier C. Ouimet R. Bergeron Y. Gravel D. (2018). Local adaptation of trees at the range margins impacts range shifts in the face of climate change. Global Ecol. Biogeogr. 27, 1507–1519. doi: 10.1111/geb.12829. PMID: 40046247

[B49] Sun K. Ma Q. H. Ji H. C. Wang A. Li Y. B. Sun R. J. . (2026). Plant trait spectrum and its climatic drivers across global realms and biomes. Global Planet. Change 256, 105136. doi: 10.1016/j.gloplacha.2025.105136. PMID: 38826717

[B50] Treurnicht M. Schurr F. M. Slingsby J. A. Esler K. J. Pagel J. (2021). Range-wide population viability analyses reveal high sensitivity to wildflower harvesting in extreme environments. J. Appl. Ecol. 58, 1399–1410. doi: 10.1111/1365-2664.13882. PMID: 40046247

[B51] Uusitalo R. Siljander M. Culverwell C. L. Mutai N. C. Forbes K. M. Vapalahti O. . (2019). Predictive mapping of mosquito distribution based on environmental and anthropogenic factors in Taita Hills, Kenya. Int. J. Appl. Earth Obs. Geoinf. 76, 84–92. doi: 10.1016/j.jag.2018.11.004. PMID: 38826717

[B52] Wang C. C. Geng L. N. Rodríguez-Casallas J. D. (2021). How and when higher climate change risk perception promotes less climate change inaction. J. Cleaner Prod. 321, 128952. doi: 10.1016/j.jclepro.2021.128952. PMID: 38826717

[B53] Wang J. Zhao J. Jiang L. Han X. Zhu Y. (2025). Predicting the potential suitable habitat of Solanum rostratum in China using the Biomod2 ensemble modeling framework. Plants (Basel) 14 (17), 2779. doi: 10.3390/plants14172779. PMID: 40941945 PMC12430113

[B54] Wani Z. A. Abdul Rahim P. P. Dar J. A. Lone A. N. Siddiqui S. (2025). Predicting the potential distribution of Podophyllum hexandrum Royle in the Himalaya under CMIP6 climate projections. Sci. Rep. 15, 25374. doi: 10.1038/s41598-025-10862-w. PMID: 40659760 PMC12259896

[B55] Xu W. B. Svenning J. C. Chen G. K. Chen B. Huang J. H. Ma K. P. (2018). Plant geographical range size and climate stability in China: Growth form matters. Global Ecol. Biogeogr. 27, 506–517. doi: 10.1111/geb.12710. PMID: 40046247

[B56] Yazdandoost F. Moradian S. Izadi A. Aghakouchak A. (2021). Evaluation of CMIP6 precipitation simulations across different climatic zones: Uncertainty and model intercomparison. Atmos. Res. 250, 105369. doi: 10.1016/j.atmosres.2020.105369. PMID: 38826717

[B57] Yue J. Li Z. Zuo Z. Wang Y. (2022). Evaluation of ecological suitability and quality suitability of Panax notoginseng under multi-regionalization modeling theory. Front. Plant Sci. 13, 818376. doi: 10.3389/fpls.2022.818376. PMID: 35574115 PMC9096839

[B58] Zahara K. Bibi Y. Qayyum A. Nisa S. (2019). Investigation of antimicrobial and antioxidant properties of Bidens biternata. Iranian J. Sci. Technol. Transaction A-Science 43, 725–734. doi: 10.1007/s40995-018-0564-2. PMID: 30311153

[B59] Zaman W. Ayaz A. Park S. (2025). Climate change and medicinal plant biodiversity: Conservation strategies for sustainable use and genetic resource preservation. Genet. Resour. Crop Evol. 72, 6275–6308. doi: 10.1007/s10722-025-02410-2. PMID: 30311153

[B60] Zhang X. Y. Wei H. Y. Zhang X. H. Liu J. Zhang Q. Z. Gu W. (2020). Non-pessimistic predictions of the distributions and suitability of under climate change using a random forest model. Forests 11, 62. doi: 10.3390/f11010062. PMID: 30654563

[B61] Zhang Y. B. Wang Y. Z. Zhang M. G. Ma K. P. (2014). Climate change threats to protected plants of China: An evaluation based on species distribution modeling. Chin. Sci. Bull. 59, 4652–4659. doi: 10.1007/s11434-014-0642-6. PMID: 30311153

